# KRAS modulates immune infiltration levels and survival outcomes in patients with lung adenocarcinoma

**DOI:** 10.1097/MD.0000000000036597

**Published:** 2023-12-29

**Authors:** Na Li, Yue Tian, Xin Liu, Ciming Pan, Jian Xue

**Affiliations:** a Harbin Medical University Affiliated Sixth Hospital, Harbin, China; b The First Affiliated Hospital of Heilongjiang University of Traditional Chinese Medicine, Harbin, China; c Hulin Traditional Chinese Medicine Hospital, Hulin, China; d Yunnan University of Chinese Medicine, Yunnan, China.

**Keywords:** KRAS, level of immune infiltration, LUAD, lung adenocarcinoma, RASs, tumorigenesis

## Abstract

The murine sarcoma virus oncogene (KRAS) is a key gene associated with tumorigenesis and chemotherapy resistance. However, little is known about the molecular mechanisms and immune infiltration of RASs in lung adenocarcinoma. Gene Expression Profiling Interaction Analysis was used for RASs expression analysis, and Kaplan–Meier analysis was used to analyze the potential of RASs in clinical prognosis. The effect of KRAS on immune infiltration was analyzed by TIMER. In addition, the correlation between KRAS expression and molecular mechanisms was investigated by TIMER and Cancer Single-cell State Atlas (Cancer SEA). KRAS expression levels were associated with good prognosis and tumor progression. Furthermore, KRAS expression correlates with several immune cell markers and regulates tumorigenesis. KRAS expression is involved in the regulation of multiple oncogenes and tumorigenesis, especially in the prognosis and immune infiltration of lung adenocarcinoma.

## 1. Introduction

Lung cancer, liver cancer, and gastric cancer are among the highest in morbidity and mortality among malignant tumors. Recent reports indicate that lung cancer is the most lethal malignancy in the world.^[1]^ The early clinical specificity of lung cancer patients is not obvious, the prognosis after intermediate and advanced treatment is very poor, and the 5-year survival rate is <20%.^[2]^ Lung adenocarcinoma (LUAD) is the most common type of non-small cell lung cancer, accounting for 40% of lung cancers, and it is reported that about 500,000 people die each year due to LUAD.^[3]^ LUAD has brought a huge burden to the society and hospitals, as well as economic difficulties and mental torture to patients and their families. Therefore, more effective markers are urgently needed for the early diagnosis and early intervention of this disease.

Metabolic reprogramming is one of the hallmarks of malignancy, and recent studies have shown that the metabolic properties and preferences of tumors change during cancer progression.^[4]^ Relevant studies have shown that the murine sarcoma virus oncogene (KRAS) plays a key role in coordinating the reprogramming of tumor metabolism in tumorigenesis, and the emerging role of KRAS oncogene driving tumor metabolism changes has attracted much attention.^[5]^ KRAS, HRAS, and NRAS genes belong to the same family of RASs, however, KRAS mutations are the most common in tumors, and KRAS mutations occur in about 30% of human tumors.^[6]^

Evidence has shown that KRAS is involved in various biological processes during carcinogenesis, such as proliferation, differentiation, apoptosis, and chemoresistance.^[7]^ In the past, KRAS was considered unlikely to be involved in cancer development, but in recent years evidence has shown that the development of many cancers depends on the continued expression and signaling of KRAS. At present, direct evidence has been found in pancreatic cancer, colorectal cancer, and other studies, and preliminary studies have also been carried out in lung cancer. The RASs family, especially KRAS, is closely related to the prognosis of patients with LUAD. However, the relationship between the underlying molecular mechanism remains unknown.

## 2. Methods and materials

### 2.1. Transcriptional expression of uncoupling proteins

Comparison of KRAS expression in tumor tissues and normal tissues was formed with FPKM of mRNAs in the GEPIA database (Gene Expression Profiling Interactive Analysis) (http://gepia.cancer-pku.cn/).^[8]^ Pan-cancer analysis was performed with the Pan-Cancer database (Assistant for clinical Bioinformatics) (https://www.aclbi.com/static/index.html#/pan_cancer).

### 2.2. The prognostic value of KRAS in patients with lung adenocarcinoma

The Kaplan–Meier Plotter (www.kmplot.com) is a gene expression online tool for assessing cancer survival to assess the prognostic significance of KRAS mRNA in patients with LUAD. KRAS genes were entered into the database to draw survival curves, and log-rank *P* values were calculated on the web page.^[9]^

### 2.3. Immune analysis of KRAS in patients with lung adenocarcinoma

The Tumor Immunity Estimation Resource (TIMER, https://cistrome.shinyapps.io/timer/) was used to comprehensively analyze the correlation of immune infiltration in KRAS in LUAD patients.^[10]^ To consolidate the analysis results, the association of KRAS and immune cell marker genes was further analyzed using the relevant modules in the TIMER platform.

### 2.4. Analysis of single cell functional modules

CancerSEA (http://biocc.hrbmu.edu.cn/CancerSEA/)^[11]^ is a database for studying different functional states at the single-cell level. Single-cell sequencing data in the CancerSEA database are from the Sequence Read Archive (https://www.ncbi.nlm.nih.gov/sra), the GEO database, and ArrayExpress Database (https://www.ebi.ac.uk/arrayexpress/). The database contains 41,900 cancer single-cell data from 25 cancers, with a total of 280 cell groups defined. There are a total of 14 functional states in the database, including angiogenesis, apoptosis, cell cycle, differentiation, epithelial-mesenchymal transition (EMT), hypoxia, inflammation, invasion, metastasis, proliferation, quiescence, and stemness. Therefore, we used the CancerSEA database to identify KRAS family-related functional states.

### 2.5. Statistical analysis

The data obtained from the above databases were statistically analyzed. Means of KRAS expression were compared using 1-way ANOVA and Student *t* test. Kaplan–Meier analysis and log-rank test were used for overall survival analysis of LUAD patients. Spearman correlation coefficient was used to evaluate the correlation between KRAS and marker genes and immune infiltrating cells. *P* < .05 was statistically significant.

## 3. Results

### 3.1. Expression of KRAS in lung adenocarcinoma

**Figure 1.**
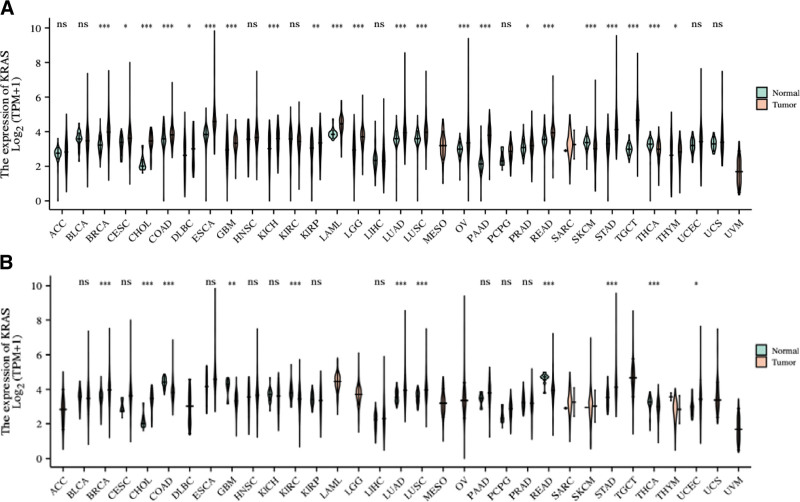
(A) KRAS mRNA expression in different tumor samples paired with normal tissue. In the figure, the normal group and the tumor group are shown by a violin diagram. *, **, *** indicate statistical significance and “ns” means not statistically significant. We found that the expression of KRAS was statistically significant in normal tissues and LUAD. (B) KRAS expression between tumor and non-tumor matched GTEx normal tissues. Violin plots of KRAS expression between tumor and non-tumor-matched GTEx normal tissues are shown, *, **, *** indicate statistical significance, and “ns” indicates no statistical significance. Statistically significant in LUAD.

KRAS is expressed in both cancer and normal lung and liver tissues, and we found that the expression of KRAS mRNA in lung tissue is higher than that in liver tissue. The right side shows normal human tissue, and the green color shows KRAS expression in different organs. The left shows the expression of red KRAS in the organs of tumor patients, with darker color representing denser distribution (Figure S1, Supplemental Digital Content, http://links.lww.com/MD/L48). KRAS mRNA levels in different tumor samples paired with normal tissues and KRAS expression between tumor and non-tumor-matched GTEx normal tissues were not significantly different (Fig. 1A and B).

### 3.2. RASs mRNA expression and prognostic value in LUAD patients

mRNA levels of RASs (KRAS, HRAS, and NRAS) in LUAD patients with different tumor stages. As shown in Figure S2, Supplemental Digital Content, http://links.lww.com/MD/L49 (In the mRNA levels of KRAS, HRAS, and NRAS in LUAD patients with different tumor stages, we found that only T1 and T2 of NRAS were statistically significant.), KRAS and HRAS were not significantly different at different stages, however, NRAS was significantly different at T1 and T2 stages (*P* < .05).

To determine the prognostic value of RASs, the Kaplan–Meier Plotter tool was used to explore the correlation between mRNA levels of RASs and survival in LUAD patients. Log-rank test analysis showed that elevated KRAS mRNA levels were significantly associated with improved overall survival (OS), disease specific survival (DSS), and progression-free interval (PFI) (*P* < .05), while HRAS, NRAS mRNA levels were not correlated with improved OS, DSS, and PFI (*P *> .05), see Figure S3A–C, Supplemental Digital Content, http://links.lww.com/MD/L50, http://links.lww.com/MD/L51, http://links.lww.com/MD/L52.

OS, DSS, and PFI. KRAS mRNA levels were statistically significant for OS, DSS, and PFI in both low- and high-expressing tissues. HRAS mRNA levels were statistically significant for OS, DSS, and PFI in both low- and high-expressing tissues. NRAS mRNA levels were statistically significant for OS, DSS, and PFI in both low- and high-expressing tissues.

### 3.3. KRAS-related clinical significance in LUAD patients—baseline data sheet

This study obtained data on patients with KRAS-related LUAD through the Clinical Significance - Baseline Information Table in Xiantao Academic (https://bioinfomatics.xiantao.love). Data source: TCGA (https://portal.gdc.cancer.gov/) RNAseq data in level 3 HTSeq-FPKM format from the LUAD (Lung Adenocarcinoma) Project (Table S1, Supplemental Digital Content, http://links.lww.com/MD/L56).

### 3.4. The relationship between KRAS mRNA level and age, gender, and smoking in patients with LUAD

At the same time, the correlation analysis of patients with different stages of LUAD, age, gender, smoking, etc was carried out. The results showed that age >65 years, male sex, and smoking were associated with KRAS mRNA levels in LUAD patients in the subgroup analysis, with statistical significance (*P* < .05) (Figure S4, Supplemental Digital Content, http://links.lww.com/MD/L53). High expression of KRAS was associated with better survival in LUAD patients with patient age, gender, and smoking.

### 3.5. The relationship between KRAS mRNA level and radiotherapy and chemotherapy in patients with LUAD

Postoperative radiotherapy and chemotherapy were independently associated with worsening of overall survival.^[12]^ This study further analyzed the correlation between radiotherapy and chemotherapy and KRAS in LUAD patients. The results showed that the hazard ratio of radiotherapy and KRAS was 1.95 (95% CI: 1.12–3.31), *P* = .016; the hazard ratio of chemotherapy and KRAS was 0.4 (95% CI: 0.24–0.66), *P* = .00021 (Figure S5, Supplemental Digital Content, http://links.lww.com/MD/L54). High expression of KRAS is associated with better survival in LUAD patients treated with radiotherapy and chemotherapy.

### 3.6. Correlation between KRAS expression and immune marker genes

The correlation between KRAS and some marker genes of immune cells, including tumor-associated macrophages (TAM), M2 macrophages, B cells, and T cells, was analyzed by TIMER. Correlation coefficients >0.2 and <–0.2 were considered meaningful and significant correlations at *P* < .05.^[13]^ Specifically, KRAS was significantly negatively correlated with T cell marker genes CD3E and CD3D, monocyte marker gene CD14, and TAM marker genes CCL5 and CD68. M2 marker gene CD163 was positively correlated with T cell marker gene CD3G (Table S2, Supplemental Digital Content, http://links.lww.com/MD/L57).

### 3.7. The relationship between KRAS mRNA level and LUAD tumor functional status

In addition, the correlation between KRAS mRNA expression level and LUAD functional status was analyzed using CancerSEA database, and the results showed that KRAS associated with invasion (*R* = 0.34, *P* = .00), hypoxia (Hypoxia) (*R* = 0.35, *P* = .00), Differentiation (*R* = 0.36, *P* = .00), EMT (*R* = 0.48, *P* = .00), Angiogenesis (*R* = 0.49, *P* = .00), Quiescence (*R* = 0.49, *P* = .00), and Metastasis (*R* = 0.65, *P* = .00) were positively correlated (Figure S6A–G, Supplemental Digital Content, http://links.lww.com/MD/L55).

Correlation analysis between KRAS and functional status of cancer cells. (A) Scatter plot showed the correlation between the UCP1 and invasion. (B) Scatter plot showed the correlation between the UCP1 and Hypoxia. (C) Scatter plot showed the correlation between the UCP1 and Differentiation. (D) Scatter plot showed the correlation between the UCP1 and EMT. (E) Scatter plot showed the correlation between the UCP1 and Angiogenesis. (F) Scatter plot showed the correlation between the UCP1 and Quiescence. (G) Scatter plot showed the correlation between the UCP1 and Metastasis.

## 4. Discussion

The development of LUAD is slow and difficult to detect, and the clinical symptoms in the early stage are not typical. Most LUAD patients are diagnosed in the middle and late stages, and most patients have extensive invasion and lymph node metastasis. Effective treatment measures.^[14,15]^ KRAS has attracted much attention in LUAD, and there are related reports. However, there is no comprehensive analysis yet. This study systematically analyzed the OS, DSS, and PFI of RASs and LUAD patients. The results showed that only KRAS was correlated. There was no significant correlation between HRAS and NRAS.^[16]^ It is worth noting that NRAS is involved in tumor T1 and T2 stages, therefore, NRAS may have diagnostic value in the early stage of LUAD, which deserves further study.

There is increasing evidence that KRAS mutations are associated with the development of many cancers, and nearly 67% to 90% of pancreatic cancer patients have KRAS mutations.^[17,18]^ KRAS mutation is close to 24.2% in patients with Hepatic cell carcinoma with extrahepatic metastasis.^[19]^ KRAS mutations are found in nearly 50% of colorectal cancer patients and are higher in women than in men.^[20]^

In this study, the clinical characteristics of LUAD patients were analyzed, and it was found that KRAS gene mutations were more common in men, people over 65 years old, and people with a history of smoking, which was consistent with the results of previous studies.^[21]^ Dual-drug chemotherapy with nonspecific cytotoxic drugs is still the main treatment modality for patients with KRAS-mutated advanced lung cancer. There is no consistent conclusion about the effect of KRAS mutation subtypes on the prognosis of chemotherapy patients.^[22]^ The results of the study showed that high KRAS mutation expression worsened survival with radiotherapy, but significantly improved with chemotherapy.

There is increasing evidence that KRAS expression is correlated with immune marker genes, KRAS is significantly negatively correlated with T cell marker genes CD3E and CD3D, monocyte marker gene CD14, TAM marker genes CCL5 and CD68, and M2 marker gene CD163. It was positively correlated with the T cell marker gene CD3G. More evidence points that immune infiltration is closely related to the proliferation, migration, and invasion of LUAD and chemotherapy resistance. KRAS is positively correlated with invasion, hypoxia, differentiation, epithelial-mesenchymal transition, angiogenesis, Quiescence, metastasis, etc. Therefore, the analysis of immune infiltration data is consistent with the conclusions of the survival analysis.

Some limitations of this study should be pointed out. The results of this study showed that there was no statistical significance in different pathological subgroups, and the NRAS in the RASs family was different at T1 and T2 stages, but not correlated in survival analysis and other aspects. Therefore, further attention needs to be paid to the clinical significance of its combined detection. In conclusion, increased KRAS expression was associated with better prognosis and increased levels of immune infiltration in LUAD. In addition, KRAS is involved in the regulation of multiple oncogenes and tumorigenesis. Our findings suggest that KRAS may serve as a potential prognostic biomarker for LUAD in the future.

## Author contributions

**Conceptualization:** Na Li, Ciming Pan.

**Data curation:** Na Li, Ciming Pan.

**Formal analysis:** Na Li.

**Funding acquisition:** Yue Tian.

**Investigation:** Yue Tian, Jian Xue.

**Methodology:** Xin Liu, Jian Xue.

**Project administration:** Xin Liu.

## Supplementary Material




















