# Supplementary figures and images for: KRAS modulates immune infiltration levels and survival outcomes in patients with lung adenocarcinoma
Source: Medicine (Baltimore). 2023 Dec 29;102(52):e36597. doi: 10.1097/MD.0000000000036597 (PMC10754580; doi:10.1097/MD.0000000000036597)

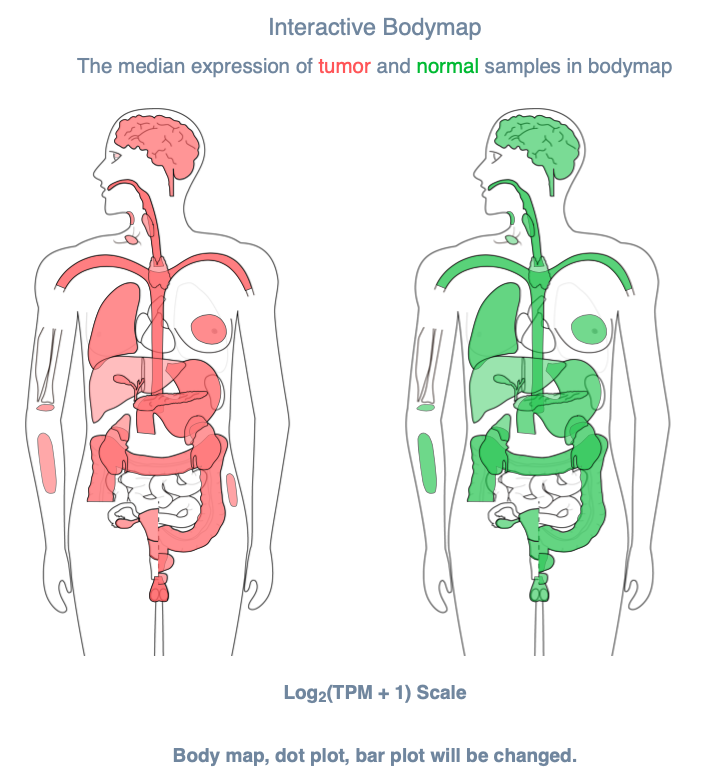

Supplement: Supplementary file 1 [file medi-102-e36597-s001.docx]

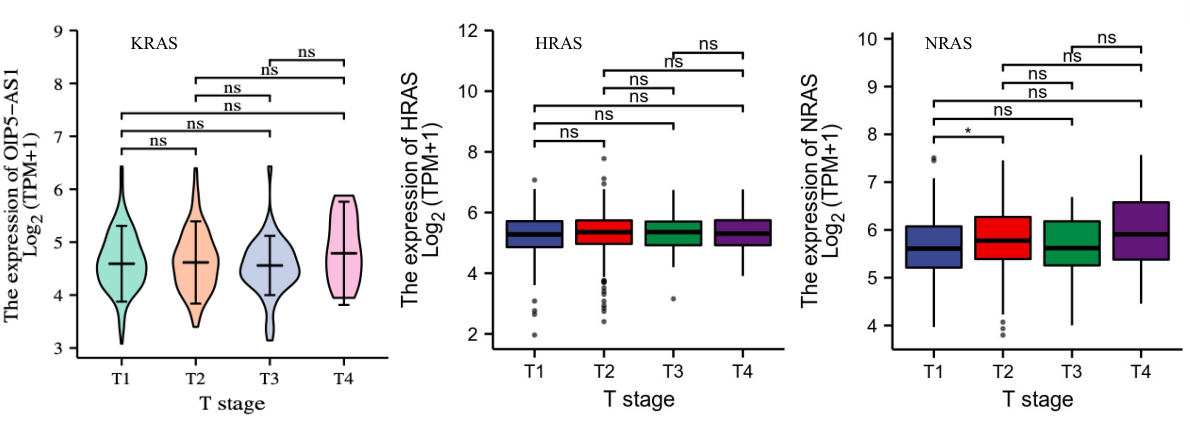

Supplement: Supplementary file 2 [file medi-102-e36597-s002.docx]

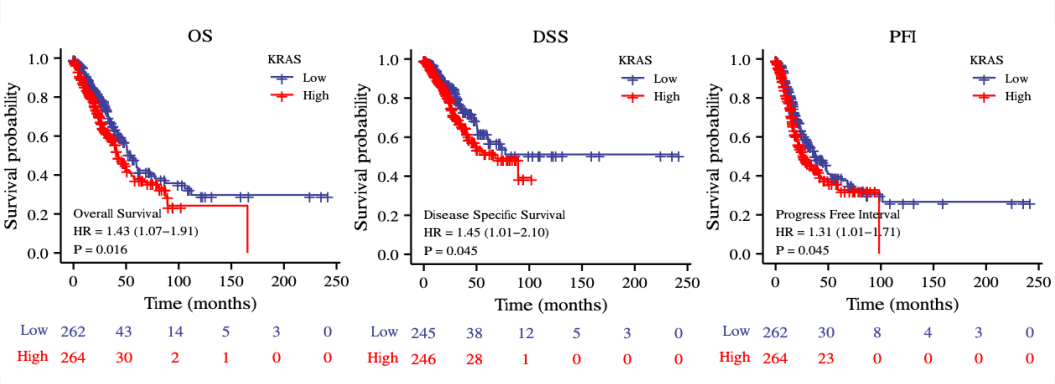

Supplement: Supplementary file 3 [file medi-102-e36597-s003.docx]

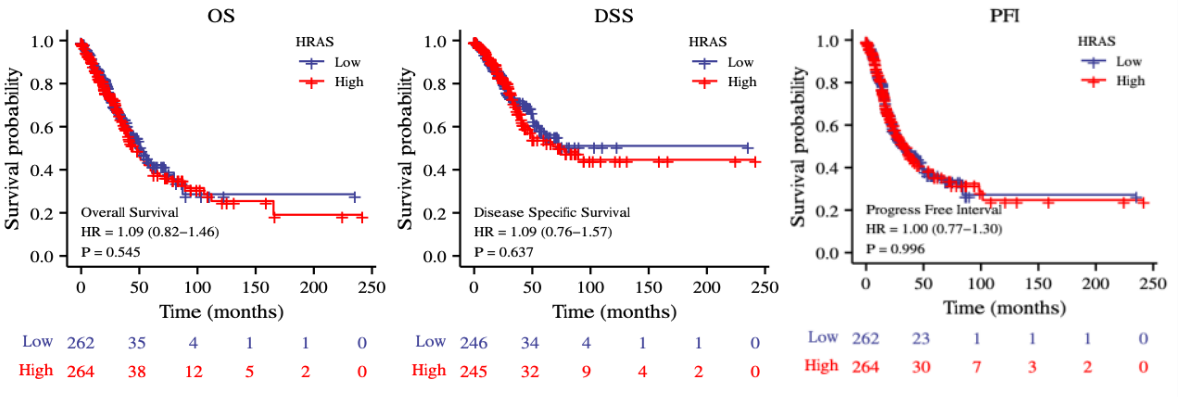

Supplement: Supplementary file 4 [file medi-102-e36597-s004.docx]

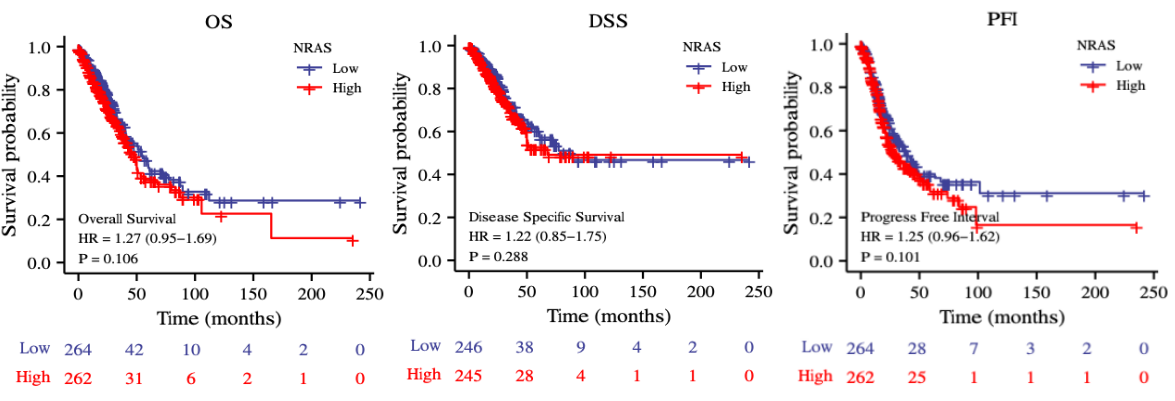

Supplement: Supplementary file 5 [file medi-102-e36597-s005.docx]

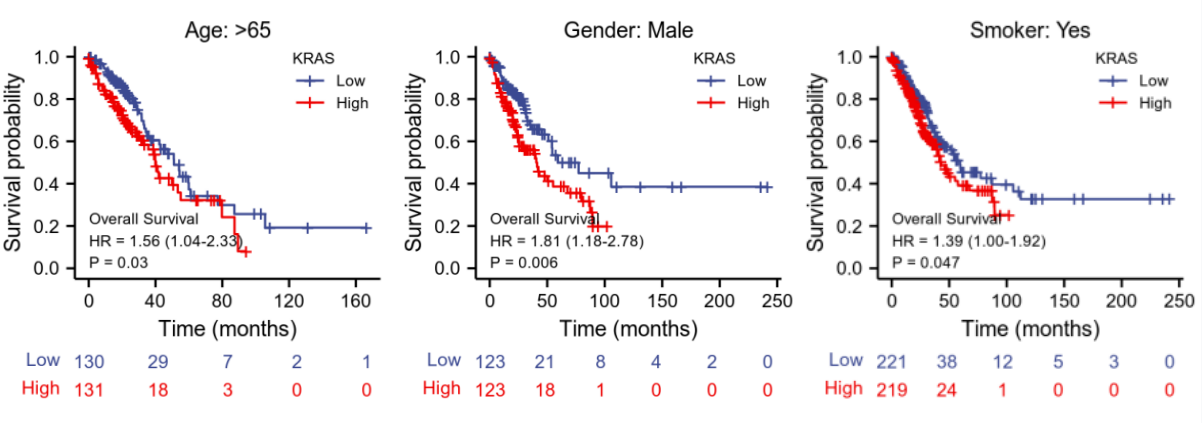

Supplement: Supplementary file 7 [file medi-102-e36597-s007.docx]

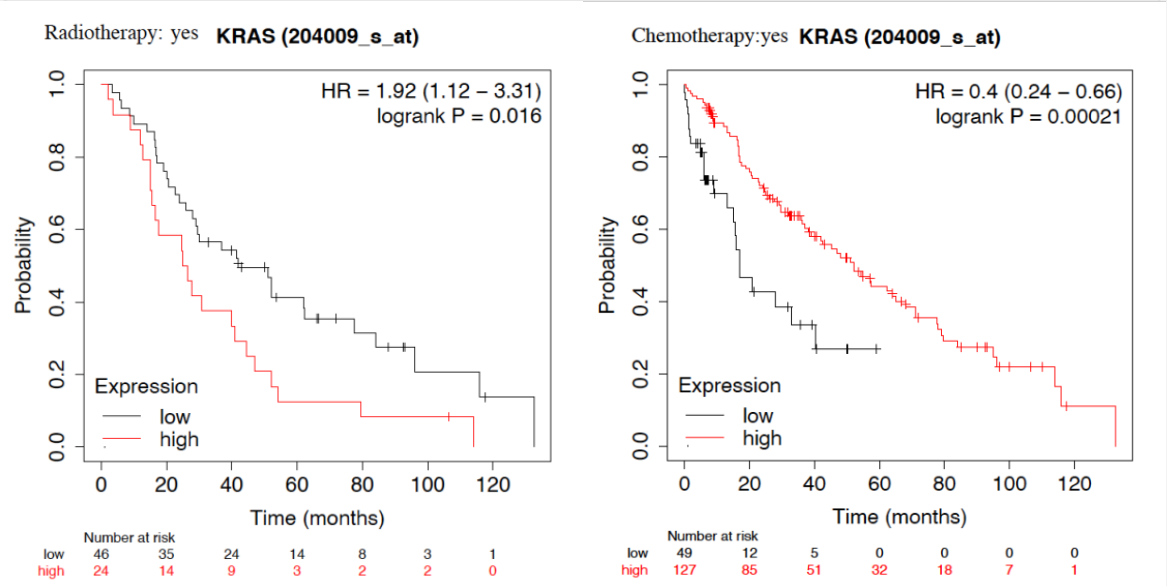

Supplement: Supplementary file 8 [file medi-102-e36597-s008.docx]

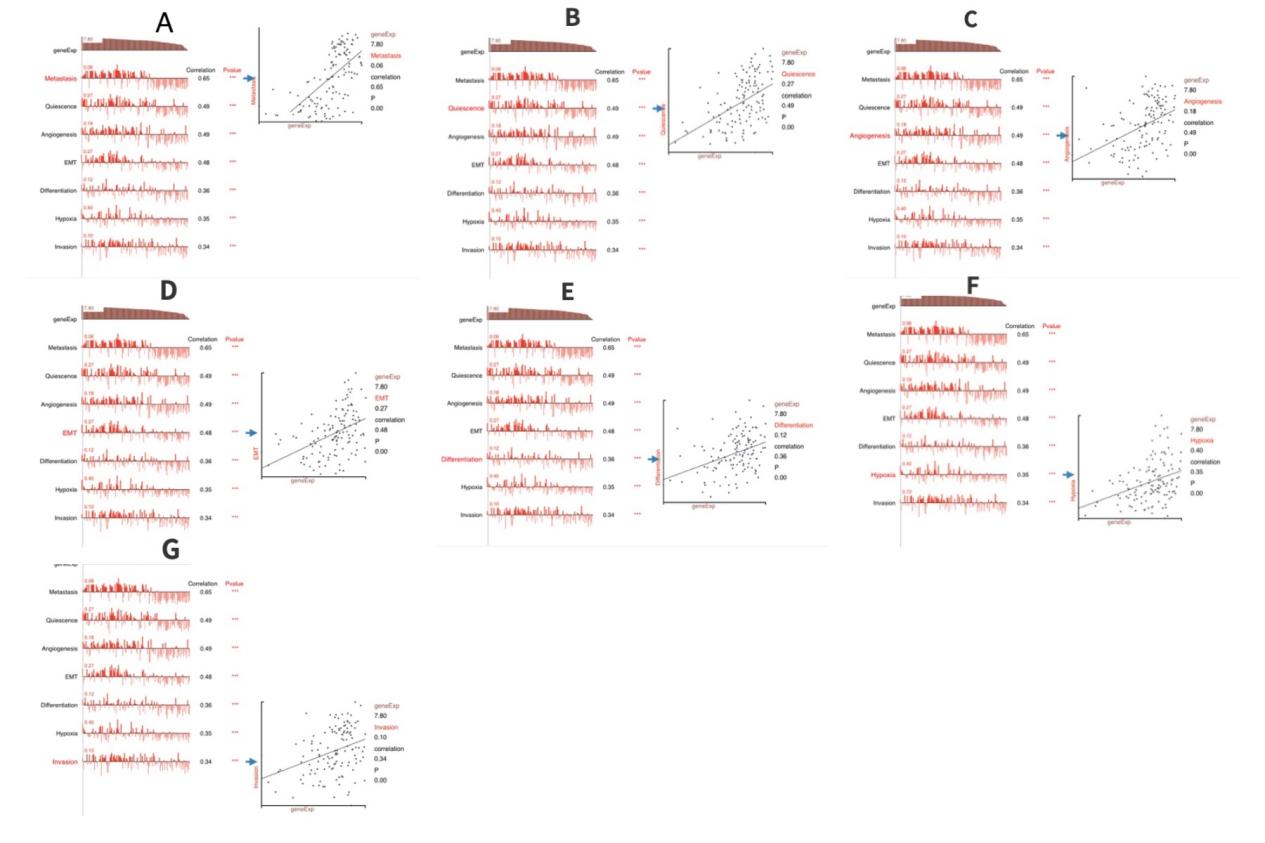

Supplement: Supplementary file 10 [file medi-102-e36597-s010.docx]
