# Supplementary material for: KRAS modulates immune infiltration levels and survival outcomes in patients with lung adenocarcinoma
Source: Medicine (Baltimore). 2023 Dec 29;102(52):e36597. doi: 10.1097/MD.0000000000036597 (PMC10754580; doi:10.1097/MD.0000000000036597)
Supplement: Supplementary file 6 [file medi-102-e36597-s006.docx]

| **Characteristic** | **Low expression of KRAS** | **High expression of KRAS** | **p** |
| --- | --- | --- | --- |
| n | 267 | 268 |  |
| T stage, n (%) |  |  | 0.049 |
| T1 | 102 (19.2%) | 73 (13.7%) |  |
| T2 | 131 (24.6%) | 158 (29.7%) |  |
| T3 | 22 (4.1%) | 27 (5.1%) |  |
| T4 | 9 (1.7%) | 10 (1.9%) |  |
| N stage, n (%) |  |  | 0.117 |
| N0 | 164 (31.6%) | 184 (35.5%) |  |
| N1 | 55 (10.6%) | 40 (7.7%) |  |
| N2 | 39 (7.5%) | 35 (6.7%) |  |
| N3 | 2 (0.4%) | 0 (0%) |  |
| M stage, n (%) |  |  | 0.872 |
| M0 | 174 (45.1%) | 187 (48.4%) |  |
| M1 | 13 (3.4%) | 12 (3.1%) |  |
| Age, meidan (IQR) | 65 (59, 72) | 66 (59, 73) | 0.475 |
