# Supplementary material for: KRAS modulates immune infiltration levels and survival outcomes in patients with lung adenocarcinoma
Source: Medicine (Baltimore). 2023 Dec 29;102(52):e36597. doi: 10.1097/MD.0000000000036597 (PMC10754580; doi:10.1097/MD.0000000000036597)
Supplement: Supplementary file 9 [file medi-102-e36597-s009.docx]

| **Description** | **Gene markers** | **Spearman’s Correlation** | **P value** |
| --- | --- | --- | --- |
| B cell | CD19 | -0.214 | 0.000 |
| T Cell | CD3E | -0.086 | 0.047 |
|  | CD3D | -0.126 | 0.003 |
|  | CD3G | 0.093 | 0.031 |
| monocyte | CD86 | 0.061 | 0.157 |
|  | CD14 | -0.102 | 0.018 |
| TAM | CCL5 | -0.117 | 0.007 |
|  | CCL20 | 0.151 | 4.68e-04 |
|  | CD47 | 0.094 | 0.029 |
|  | CD68 | -0.077 | 0.077 |
| M2 | CD163 | 0.143 | 9.14e-04 |
| GATA3 | GATA3 | 0.035 | 0.421 |
